# A Pretraining-Retraining Strategy of Deep Learning Improves Cell-Specific Enhancer Predictions

**DOI:** 10.3389/fgene.2019.01305

**Published:** 2020-01-08

**Authors:** Xiaohui Niu, Kun Yang, Ge Zhang, Zhiquan Yang, Xuehai Hu

**Affiliations:** College of Informatics, Hubei Key Laboratory of Agricultural Bioinformatics, Huazhong Agricultural University, Wuhan, China

**Keywords:** deep learning, pretraining, retraining, tissue-specific enhancers, prediction

## Abstract

Deciphering the code of cis-regulatory element (CRE) is one of the core issues of today’s biology. Enhancers are distal CREs and play significant roles in gene transcriptional regulation. Although identifications of enhancer locations across the whole genome [discriminative enhancer predictions (DEP)] is necessary, it is more important to predict in which specific cell or tissue types, they will be activated and functional [tissue-specific enhancer predictions (TSEP)]. Although existing deep learning models achieved great successes in DEP, they cannot be directly employed in TSEP because a specific cell or tissue type only has a limited number of available enhancer samples for training. Here, we first adopted a reported deep learning architecture and then developed a novel training strategy named “pretraining-retraining strategy” (PRS) for TSEP by decomposing the whole training process into two successive stages: a pretraining stage is designed to train with the whole enhancer data for performing DEP, and a retraining strategy is then designed to train with tissue-specific enhancer samples based on the trained pretraining model for making TSEP. As a result, PRS is found to be valid for DEP with an AUC of 0.922 and a GM (geometric mean) of 0.696, when testing on a larger-scale FANTOM5 enhancer dataset *via* a five-fold cross-validation. Interestingly, based on the trained pretraining model, a new finding is that only additional twenty epochs are needed to complete the retraining process on testing 23 specific tissues or cell lines. For TSEP tasks, PRS achieved a mean GM of 0.806 which is significantly higher than 0.528 of gkm-SVM, an existing mainstream method for CRE predictions. Notably, PRS is further proven superior to other two state-of-the-art methods: DEEP and BiRen. In summary, PRS has employed useful ideas from the domain of transfer learning and is a reliable method for TSEPs.

## Introduction

One of the core issues of today’s biology is to decipher the code of cis-regulatory element (CRE) (Yáñez-Cuna et al., 2013). Enhancers are important distal CREs and play significant roles in gene transcriptional regulation (Bulger and Groudine, 2011). The regulation of gene expression by enhancers acts as a binding platform for recruiting transcriptional factors and cofactors to activate transcriptions of target genes (Shlyueva et al., 2014; Li et al., 2016).

Accurate identification of enhancer locations across the whole human genome is extremely important and is currently of great interest based on two facts: (1) ENCODE project indirectly identified >500,000 putative enhancers (Hoffman et al., 2012; Ernst and Kellis, 2012) and their total length might reach 12% of the human genome (Fishilevich et al., 2017), suggesting the enhancer element is a nonnegligible component of the human genome, and (2) genome-wide association studies (GWAS) in the past decade locked over 55% of the disease-associated SNPs in the non-coding DNA (Maurano et al., 2012). Some of them were reported to be exactly located within the enhancer regions, implying strong relationships between human diseases and the enhancer element. For example, a cancer-associated SNP of rs6983267 identified by human GWAS of intestinal tumors was reported to be contained within a Myc enhancer regulatory element (Sur et al., 2012). However, because of two hallmarks of enhancers, it is a challenging problem to distinguish them from other CREs: regulating manners of long-distance and bidirectionality. Typically, distal enhancers are located more than 10kb away from the target genes they regulate (Bulger and Groudine, 2011), and on the other hand, an enhancer can bidirectionally function both at the upstream and downstream of the target gene, which doubles the searching difficulty (Li et al., 2016).

In the past two decades, researchers have developed several distinct experimental strategies from different viewpoints for inferring the locations of active enhancers, such as transgenic mouse assay (Visel et al., 2007), using chromatin features from ENCODE data (Heintzman et al., 2009; Ernst and Kellis, 2012; Hoffman et al., 2012), massively parallel report assay (MPRA) employing barcode-containing transcripts (Melnikov et al., 2012; Kwasnieski et al., 2014; Shen et al., 2015), STARR-seq using self-transcribing transcripts (Arnold et al., 2013), and cap analysis of gene expression (CAGE), utilizing enhancer RNA (eRNA) (Andersson et al., 2014).

An alternative way for identifying enhancers is by computational methods, which try to learn intrinsic features from credible enhancer sequence samples and then build reliable prediction models for making evaluation and discovery. This mechanistic approach is feasible because DNA sequence is both sufficient and necessary for enhancer activity: (1) an enhancer sequence can still drive gene expressions when being removed from its endogenous context to upstream of a reporter gene (Kvon et al., 2012), suggesting its sufficiency; (2) a disruption of core motif within an enhancer sequence would substantially reduce enhancer activity (Kwasnieski et al., 2014), implying its necessity. As a matter of fact, a series of studies have already addressed this issue in the past decade (Lee et al., 2011; Kleftogiannis et al., 2014; Liu et al., 2016; Beer, 2017; Yang et al., 2017). A pioneer finding is that *k*-mer features of length 6 are predictive sequence features for discriminative enhancer prediction (DEP) when using ChIP-seq data of P300 (Lee et al., 2011). An advanced version of *k*-mer tool named gkm-SVM, which is one of the most popular method for regulatory sequence predictions (Ghandi et al., 2014), was recently employed for DEP (Beer, 2017). iEnhancer-2L proposed to use pseudo *k*-tuple nucleotide composition features for identifying enhancers and their strengths (Liu et al., 2016). Notably, BiRen (Yang et al., 2017) recently introduced more advanced tools including convolutional neural network (CNN) and bidirectional recurrent neural network (BRNN) for DEP. The above methods were all developed for DEP and they would give no answers about tissue-specific enhancer prediction (TSEP). At this point, DEEP (Kleftogiannis et al., 2014) integrated three resources of enhancer data, ENCODE, FANTOM5, and VISTA, and developed an ensemble model for DEP as well as for TSEP.

Although deep learning methods including BiRen were adopted for DEP, they have some problems that should be addressed for the task of TSEP. In the past 5 years, deep learning tools were successfully applied in some areas of biology from genomics and imaging to electronic medical records (Webb, 2018). Particularly, CNN has become a dominating method in various prediction problems, including predicting transcriptional factor binding sites (TFBS) (Alipanahi et al., 2015; Quang and Xie, 2016; Zeng et al., 2016) and predicting chromatin effects of DNA variants (Zhou and Troyanskaya, 2015; Kelley et al., 2016; Liu et al., 2018; Min et al., 2017). However, these successful experiences might not be directly transferred to TSEP by the following dilemma: on the one hand, a given enhancer for one specific tissue might not be activated in another tissue, so it is impossible to make multiple TSEPs only with one deep learning model; on the other hand, if we divide the whole enhancer dataset into multiple tissue-specific enhancer datasets and then build multiple prediction models, the sample size of each tissue is only several hundred or a few thousands, which is far less than the number of parameters (often hundreds of thousands) needed to be trained, suggesting that the built models might take high risks of falling into overfitting.

Here, we proposed a novel deep learning training strategy named pretraining-retraining strategy (PRS), which is especially appropriate for the task of TSEP. To address the problem of multiple TSEPs, we decomposed the training process into two successive stages: a pretraining stage and a retraining stage. The pretraining stage is designed for learning an appropriate network structure with optimal model hyperparameters of one model by using the whole enhancer data. Subsequently, a retraining stage is adopted only with a given tissue-specific enhancer dataset based on the trained pretraining model, suggesting a novel training pattern of one pretraining model together with multiple retraining models. To address the problem of overfitting, PRS allows all the hyperparameters to learn reasonable values when the pretraining stage is completed. And those reasonable values are good initial values of the retraining process, which enable the retraining model to converge very fast even with limited number of tissue-specific enhancer samples. PRS was tested on FANTOM5 enhancer data and was proven to be a powerful model for TESP.

## Materials and Methods

### Datasets Preparation

In this work, the FANTOM5 enhancer data was used for performing prediction tasks. FANTOM consortium released a large-scale enhancer dataset that contains 65,423 enhancer activities (measured by TPM (tag per million mapped reads) of their expressions of eRNA) in 1,829 distinct tissues or cell lines in human (Andersson et al., 2014), which was recorded as a matrix *E*_65423×1829_ with 65,423 rows and 1,829 columns (http://fantom.gsc.riken.jp/5/datafiles/latest/extra/Enhancers/).

In the pretraining stage, we used the following strategy for constructing a large-scale enhancer dataset: at first, we took a cut-off criterion of *TPM_min_* ≥ 0.08 (presents the minimal nonzero value of TPM across all tissues and cell lines of a given enhancer) to select most active enhancers, leaving only 5386 enhancers passing this criterion. Secondly, we excluded enhancers shorter than 100bp and fixed the enhancer sequence length at 1000bp with 4667 enhancers. Finally, we employed a redundancy reduction procedure CD-HIT (Huang et al., 2010) with a cutoff threshold of 0.8 and 4653 enhancers were remaining as the final positive samples. The length distribution of all 4,653 enhancer positive samples can be found in **Supplementary Figure 1**. We randomly selected 46,530 DNA sequences with length of 1,000 bp as negative samples from non-enhancer intergenic regions (obtained from the GRCh37 reference genome by excluding exon, intron and known enhancers) to meet a consensus of recent studies (Kleftogiannis et al., 2014; Liu et al., 2016; Yang et al., 2017).

In the retraining stage, 23 representative tissues or cell lines were chosen for showing cell-specific enhancer prediction performances. We also took a cut-off criterion of TPM 0.8.0.8 is the 75% quantile of the whole TPM distribution, implying that the condition of larger than 0.8 guarantees activity of enhancer) to select most active enhancers for each tissue or cell line. Ten times of the amount of each positive sample were selected as the corresponding negative samples.

### Learning Subsequence Features With CNN

**Figure 1:**
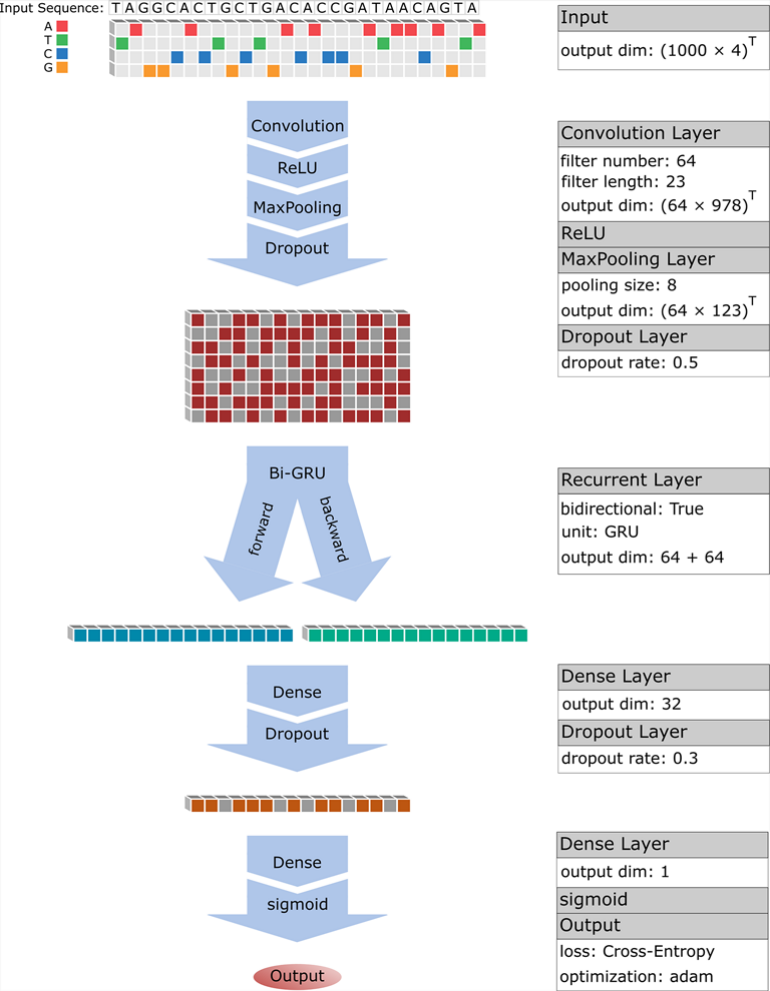
Flow chart of hybrid deep learning architecture.

CNN is a modern combination of convolutional operator and classic neural network by introduction of some advanced techniques including rectified linear unit (ReLU), pooling and dropout. Convolutional operator is very powerful for detecting significant local features that are further denoised by ReLU and pooling. When performing prediction with neural network, CNN was proven efficient and successful in various image recognition tasks including handwriting recognition, face recognition (LeCun et al., 2015). Here, we adopted a similar framework with DeepBind (Alipanahi et al., 2015) to perform CNN model, which in turn includes three layers: a convolution layer (Conv), a activation layer (ReLU), a pooling layer (Pool), where the outputs of the final layer are regarded as selected features of the inputs (**Figure 1**).

### Learning Dependencies With Bidirectional GRU

Recurrent neural network (RNN) is one kind of the advanced ANN model that has a “memory” which could capture the previous information, which is appropriate to analyze the sequential data (Schuster and Paliwal, 1997). Over the years, more advanced architectures of RNNs were developed to overcome shortcomings of the classic RNN model. Among them, bidirectional RNN (BRNN) is designed for those situations where output at time step is not only associated with the previous states, but also with future information. Because of the forward and inverse strand in enhancer sequences with bidirectional regulation function, BRNN model was proven to be very efficient to deal with regulatory sequence prediction problems (Quang and Xie, 2016).

However, BRNN still suffers a vanishing gradient problem that makes it hard to capture the long-term dependencies in the sequential data. For solving this problem, a gated recurrent units (GRU) was proposed by Bahdanau et al. (2014) by introducing some new concepts including update gate, reset gate and candidate “memory” layer. In this study, the bi-directional gated recurrent unit (Bi-GRU) was designed to connect with the last layer of CNN (the dropout layer) and six matrices WU will be learned by data (**Figure 1**).

### Model Design and PRS

Previous studies on TFBS predictions reported that the convergent filter matrices of the CNN layer are exactly consistent with TF binding motif (Alipanahi et al., 2015; Zhou and Troyanskaya, 2015; Kelley et al., 2016; Quang and Xie, 2016), suggesting CNN is efficient for learning local subsequence features. More importantly, a recent study (Quang and Xie, 2016; Yang et al., 2017) had used RNN layer to effectively address the dependence of the adjacent features in a sequence. Here, we adopted a similar deep learning model of BiRen (Yang et al., 2017) that added an RNN layer following the CNN layer (**Figure 1**). We expect to firstly learn local subsequence features (TF motifs) of an enhancer sequence with CNN, and then to learn how to combine these motifs (dependence of motifs) to form an enhancer sequence with RNN.

To solve the problem of TSEP, we proposed a novel PRS. Our idea is that we firstly use the whole FANTOM5 enhancer data (containing all tissues and cell lines) to determine an optimal network structure and all the model parameters, based on which we construct and record the pretraining model. Theoretically, such a pretraining model is only valid for discriminating enhancer from non-enhancers. For a given tissue, we will then take a retraining strategy by redoing training process with its tissue-specific enhancer data based on the pretraining model.

### Pretraining With the Whole FANTOM5 Enhancer Data

We performed a pretraining process with the whole FANTOM5 enhancer data of Enhancer4653, which contains 4653 enhancer sequences and 46530 non-enhancer sequences. Firstly, we divided the whole dataset into three portions: 10/12 as training set E_train for training model), 1/12 as validation set E_va (for determining an optimal epoch) and 1/12 as testing set E_test (for evaluating model). To begin with a CNN structure, the initial values of model hyperparameters including filter number M, filter length m and pooling size p were set to be 64, 5 and 3 respectively. Subsequently, the output of CNN is turned as the input of RNN. Finally, a neural network with 32 neurons (a weight matrix of WM) was designed to be followed with the RNN layer and the output of the neural network NN will further be processed by a sigmoid function for mapping the predicted values into interval [0,1] (**Figure 1**):

$$\hat{y}=sigmoid\left(NN\right)=\frac{1}{1+e^{-NN}}$$

which is considered as the final predicted value of each sample. This is the end of forward computation.

Here we took a rational strategy for preventing overfitting, which aims to find an optimal epoch minimizing objective _va_ as:

$${\text{objective}}_{\text{va}}={\text{crossentropy}}_{\text{va}}+\lambda_{1}{\left\|M\right\|}_{1}+\lambda_{2}{\left\|WU\right\|}_{1}+\lambda_{3}{\left\|WM\right\|}_{1},$$

$${\text{crossentropy}}_{\text{va}}=-\frac{1}{n}\sum_{y_{i}\in E\_va}\left[y_{i}\log{\hat{y}}_{i}+(1-y_{i})\log(1-{\hat{y}}_{t})\right],$$

where those *y*_i_ ∈ *E_va* belong to the validation set E_va and they never appeared in the training process. The strategy of minimizing objective _va_ not objective _train_, will effectively prevent overfitting and finally obtain the pretraining model (we call it the FANTOM model) with all the model parameters and hyperparameters determined. We finally evaluated effectiveness of the FANTOM model with predicting accuracy on all elements belonging to the testing set E_test.

### Retraining With Specific Tissue (Cell Lines) Enhancer Data

Once we have the FANTOM model, we next implement a retraining strategy to predict tissue-specific enhancer based on it. A hypothesis of the retraining strategy is that a specific tissue enhancer dataset has similar pattern with the whole FANTOM5 enhancer dataset, which implies that the predicting model of tissue-specific enhancer might share the same network structure and all the model hyperparameters of FANTOM model. The only differences between them are the updated values of those parameters including filter matrices M and weight matrix WM.

Being different from regular training process that starts with random initial parameters, our novel retraining strategy will start with the convergent values of parameters obtained in the FANTOM model. The retraining strategy has some advantages when comparing with regular training: (1) it will rapidly reach optimal prediction accuracy with only dozens of epochs, implying it is time-saving; (2) the optimal prediction accuracy will be significantly better than that of a direct training (not begin with the pretraining model).

### Evaluation of the Prediction Performance

Here, we used five indices for evaluating the prediction performance of models: sensitivity (Sens or recall), specificity (Spec), precision, accuracy (ACC), geometric mean (GM) value and Matthew’s correlation coefficient (MCC):

$$\{\begin{array}{l} Sens=recall=\frac{TP}{TP+FN},\\ Spec=\frac{TN}{TN+FP},\\ precision=\frac{TP}{TP+FP},\\ ACC=\frac{TP+TN}{TP+FP+TN+FN},\\ GM=\sqrt{precision\cdot recall}\\ MCC=\frac{TP\cdot TN-FP\cdot FN}{\sqrt{\left(TP+FN)(TP+FP)(TN+FP)(TN+FN\right)}} \end{array}$$

To test the balance between true positive and false positive rates, another evaluating index is the Area Under the ROC Curve (AUC). Because of the imbalance between the positive and negative dataset, we applied GM as an important index to assess the performance.

## Results

### Predicting Housekeeping Enhancers With the FANTOM Model

**Figure 2:**
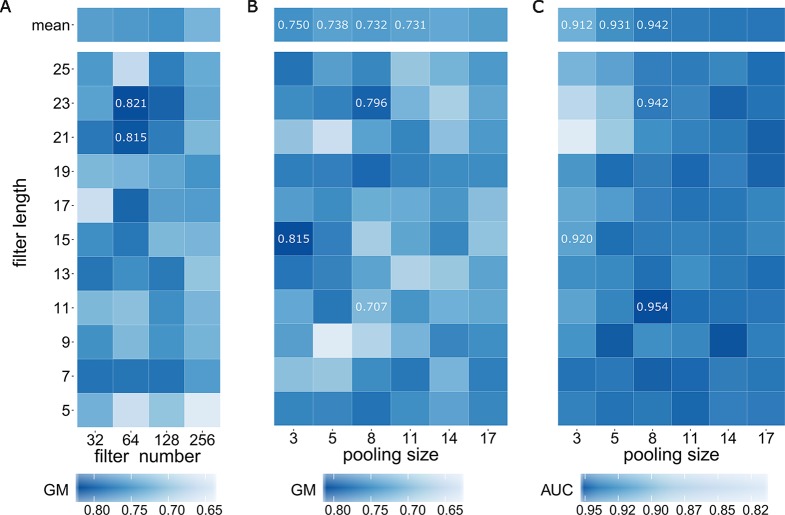
Determining optimal model hyperparameters of filter number, filter length, and pooling size. **(A)** GM values of grid search on the combinations of filter number and filter length. **(B)** GM values of grid search on the combinations of filter length and pooling size. **(C)** AUC values of grid search on the combinations of filter length and pooling size.

We first determined optimal values of three model hyperparameters including filter number M, filter length m, and pooling size p within the CNN layer with the training data E_train the validation set E_va and the testing set E_test When considering the optimal filter number, some previous works reported their choices. DeepBind (Alipanahi et al., 2015) used 16 filters for learning TF motifs; DeepSEA (Zhou and Troyanskaya, 2015) adopted three layers of CNN and took 320, 480, and 960 filters for learning chromatin features respectively; Basset (Kelley et al., 2016) employed three layers of CNN of 300, 200, 200 filters for chromatin accessibility prediction. Based on these existing experiences, we executed a parameter optimization strategy using grid search on the combinations of filter number (32, 64, 128, 256) and filter length [all odd numbers in (5, 25)] (**Figure 2**). Although researchers often used ACC or AUC value for evaluating prediction model (Liu et al., 2016; Beer, 2017; Yang et al., 2017), we here employed GM for evaluation because assessment with GM is more appropriate for extremely imbalance dataset (Kleftogiannis et al., 2014) (1:10 in this study). As a result, a maximal GM value of 0.821 was achieved at the combination of filter number of 64 and filter length of 23. Although, another high GM value of 0.815 was also achieved at the combination of filter number of 64 and filter length of 21, we finally determined the optimal filter number as 64.

After fixing filter number of 64, we then took a further grid search on the combinations of filter length with all odd numbers in [5,25] and pooling size of 3, 5, 8, 11, 14 and 17. We here employed GM value (**Figure 2**) together with AUC value (**Figure 2**) for a comprehensive evaluation. As a result, a maximal GM value of 0.815 was achieved at the combination of filter length of 15 and pooling size of 3 and the combination of filter length of 23 and pooling size of 8 achieved the second rank with GM value of 0.796. We noted that GM values exhibit a decreasing trend when pooling size is increasing (the column means of 3, 5, 8 and 11 are 0.750, 0.738, 0.732 and 0.731 respectively). In addition of the fact that larger pooling size would lose more information, we discarded the situations when pooling size is larger than 8 and only considered the situations with pooling size of 3, 5 and 8. We next focus on another evaluation indicator, AUC, for further searching. Interestingly, AUC values perpetuate an opposite trend when pooling size is increasing: the column means of 3, 5 and 8 are 0.912, 0.931 and 0.942 respectively, indicating that we should choose pooling size with 8. Although the maximal AUC value of 0.954 was achieved at filter length of 11 when fixing pooling size with 8. A comprehensive evaluation both using GM value and AUC value finally confirmed that the optimal filter length is 23 and the optimal pooling size is 8 because GM value of filter length of 11 was only 0.707 (significantly lower than 0.796 of filter length of 23).

In summary, we successively determined three important model hyperparameters as follows: filter number of 64, filter length of 23 and pooling size of 8. After confirming them, the FANTOM model was reevaluated *via* a 5-fold-cross-validation for a more objective assessment (**Table 1**). In the large-scale imbalanced enhancer dataset, the FANTOM model achieved a great AUC value of 0.922 (**Supplementary Figure 3**), an acceptable MCC value of 0.527, and an acceptable AUPRC value of 0.619 (**Supplementary Figure 2**) for this imbalanced dataset. In a word, the FANTOM model is a reliable prediction model on dataset of Enhancer4653, which consists of 4653 housekeeping enhancers (Zabidi et al., 2015) and 46530 non-enhancers, implying it has potential to be a reliable model for housekeeping enhancer prediction.

**Table 1:** Prediction performances of pretraining stage with large-scale FANTOM5 enhancer data via a five-fold-cross-validation.

Enhancer dataset	Sample size	ACC	AUC	SEN	SPE	MCC	GM
FANTOM5 enhancer data	4653 + 46530	0.929	0.922	0.499	0.972	0.527	0.696

### Predicting Tissue-Specific Enhancers With a Retraining Strategy

Next we proposed to predict tissue-specific enhancers with a retraining strategy, which aims to build an updated model based on the pretraining model when adding a given tissue-specific enhancer dataset. Similar as before, a training epoch containing a cycle of forward computation and backpropagation was adopted to perform updating.

**Figure 3:**
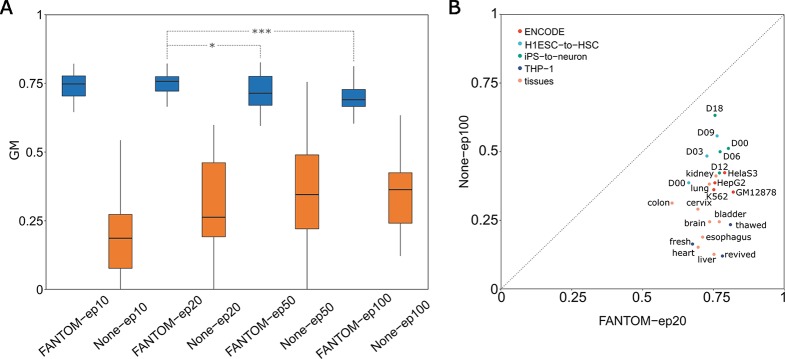
Determining optimal pretraining-retraining model and comparison with classic model with no pretraining stage. **(A)** Comparison analysis determines FANTOM-ep20 model to be the optimal pretraining-retraining model. **(B)** Comparison of GM values between FANTOM-ep20 models and None-ep100 models on 23 different tissues or cell lines.

Next two specific problems which arise are: how many epochs is at least required and how many epochs is optimal? To answer these, based on the FANTOM model, we designed four groups of retraining with four distinct numbers of epochs: 10 epochs named FANTOM-ep10, 20 epochs named FANTOM-ep20, 50 epochs named FANTOM-ep50 and 100 epochs named FANTOM-ep100. Meanwhile, we performed another four groups of *ab initio* training (not based on the FANTOM model): 10 epochs named None-ep10, 20 epochs named None-ep20, 50 epochs named None-ep50, and 100 epochs named None-ep100. Training on 23 selected groups of tissue-specific enhancer datasets (Materials and methods), a total of eight boxplots representing their GM values is given in **Figure 3**, from which we found two interesting facts: (1) GM values of four pretraining-retraining models (starting with FANTOM-) are far greater than those of *ab initio* training models (starting with None-), suggesting the importance and necessity of PRS; (2) among four pretraining-retraining models, GM values of FANTOM-ep20 are relatively higher, though no significant difference was found between FANTOM-ep20 and FANTOM-ep10 (one-sided t-test, p-value = 0.31). However, significant difference was found between FANTOM-ep20 and FANTOM-ep50 (one-sided t-test, p-value = 0.036), suggesting FANTOM-ep50 (and FANTOM-ep100) model might fall into a problem of overfitting. In a word, retraining with 10 epochs is at least required and retraining with 20 epochs might be a good choice. It is not necessary to retrain with epochs larger than 50, which is not only time-consuming but also is easy to fall into overfitting.

After determining the optimal retraining epochs as 20, let us show the superiority of FANTOM-ep20 model by precisely comparing it to None-ep100 model (the best model within None models). From **Figure 3**, it is obvious that all the points located below the line y = x, suggesting that FANTOM-ep20 model is superior to None-ep100 model at each tissue. Furthermore, 23 FANTOM-ep20 models take their GM values between 0.606 and 0.822 (with a mean of 0.746), whereas 23 GM values of None-ep100 models distribute from 0.122 to 0.634 with a mean of 0.345. A statistical t-test showed that the former is extremely greater than the latter (p-value = 1.44e-12), suggesting the difference between these two is huge. Without a pretraining stage, TSEPs using deep learning model are bad due to very low Sens values. It is widely accepted that positive sample predictions are hard when training on an extremely imbalanced dataset. The mean of 23 Sens values of None-ep100 models has a very low mean of 0.141, suggesting only 14% of positive samples were accurately predicted. By contrast, when taking PRS, 23 Sens values of FANTOM-ep20 models has a mean of 0.580, implying FANTOM-ep20 model accurately identified about 60% of positive samples. In summary, the prediction on tissue-specific enhancer will be unreliable if a pretraining stage was absent, whereas it will be much better and more acceptable by adding a pretraining stage.

We investigated the resource consumption of prediction of enhancer samples by running our script on a test computer with Ubuntu 18.04 on processors of Intel(R) Core(TM) i7-7700 CPU @ 3.60GHz, GPU of GeForce GTX 1080 Ti and 24 GB RAM. When running on 4616 testing sequences with a length of 1000 bp, a total of 1.28s was needed for such predictions, implying that the average computation time of each DNA sequence was about 2.77 × 10^-4^ second.

### Comparisons With Other Existing Methods

To further show the superiority of our method, comprehensive comparisons with three state-of-the-art methods, gkm-SVM (Ghandi et al., 2014; Ghandi et al., 2016; Beer, 2017), DEEP (Kleftogiannis et al., 2014), and BiRen (Yang et al., 2017), were performed. There are two distinct strategies for such a comparison: one is to run other tools on our dataset; the other is to run our method on existing dataset that other method used.

**Figure 4:**
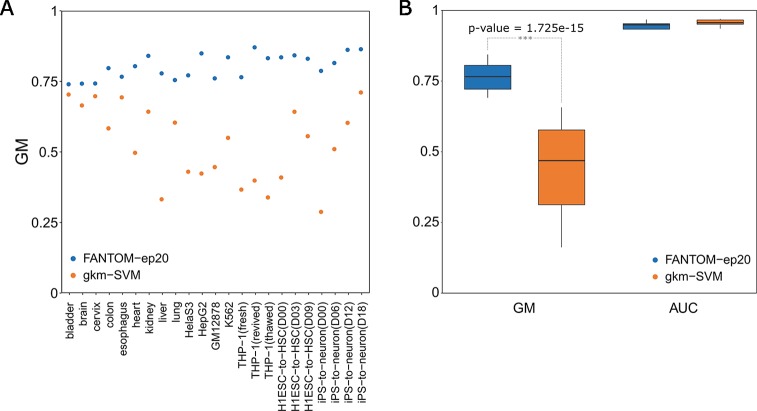
Comparisons between our FANTOM-ep20 model and gkm-SVM tool on 23 different tissues or cell lines. **(A)** One-to-one direct comparison of GM value on each tissue or cell line. **(B)** Distribution comparisons of GM values and AUC values with box plots.

We first adopted the former comparison strategy for gkm-SVM. Gkm-SVM is one of the most popular methods for regulatory sequence prediction (Ghandi et al., 2014) and has gradually become a dominating method in this area (Ghandi et al., 2016). We downloaded its R package from the website https://cran.r-project.org/web/packages/gkmSVM/index.html and then run it on our 23 tissue-specific enhancer datasets with its default parameters of L=10, K=6. A direct comparison with our best model of FANTOM-ep20 can be found in **Figure 4**, which shows the point-to-point comparisons of GM values on 23 tissues or cell lines. It is obvious that all the blue points representing those GM values (a mean of 0.806) achieved by FANTOM-ep20 models are above the orange points (a mean of 0.528) by gkm-SVM, suggesting our FANTOM-ep20 model is superior to gkm-SVM on GM values. This is further confirmed by the box-plots of these two and a t-test between them with a p-value of 1.725e-15 in **Figure 4**, though AUC values of gkm-SVM (a mean of 0.969) are slightly greater than those of our FANTOM-ep20 model (a mean of 0.957).

We next applied the later comparison strategy for DEEP and BiRen. DEEP (Kleftogiannis et al., 2014) trained many individual models for 36 different tissues from FANTOM enhancer data but it only provided the detailed prediction results on three specific tissues: heart, liver, and brain, which were chosen for comparisons. Using the latest version of FANTOM5 enhancer data, we set the cutoff thresholds with *TPM >* 1;*TPM >*4;*TMP >*1 to select three groups of tissue-specific enhancers whose numbers are closest to those numbers provided by DEEP (**Table 2**). To be consistent with DEEP, the negative samples were chosen from random intergenic regions with 10 times number of positive samples of each tissue. After performing the optimal testing strategy (40% for training and 60% for testing) of DEEP, ACC values of FANTOM-ep20 models of heart, liver, and brain were 0.946, 0.982, and 0.906, respectively, which are greater than 0.822, 0.745, and 0.853 of DEEP (**Table 2**), suggesting our model has higher prediction accuracy compared with DEEP. In their article, DEEP claimed that great superiority of their model is prediction balance on imbalance dataset, which is measured by GM value. While comparing GM values, our FANTOM-ep20 models of heart, liver and brain achieved 0.805, 0.946 and 0.766, which are comparable with 0.812, 0.741and 0.843 of DEEP respectively (**Table 2**).

**Table 2:** Comprehensive comparisons of FANTOM-ep20 model with DEEP and BiRen.

Comparison targets	Data source	Sample size	Method	ACC	AUC	Sens	Spec	MCC	GM
**DEEP**	Heart	295 + 2950	DEEP	0.822	NA	0.802	0.824	NA	0.812
		239 + 2390	FANTOM-ep20^a^	0.946	0.963	0.664	0.976	0.669	0.805
	Liver	84 + 840	DEEP	0.745	NA	0.740	0.755	NA	0.741
		75 + 750	FANTOM-ep20	0.982	0.990	0.905	0.989	0.891	0.946
	Brain	639 + 6390	DEEP	0.853	NA	0.832	0.855	NA	0.843
		619 + 6190	FANTOM-ep20	0.906	0.915	0.630	0.933	0.501	0.766
**BiRen**	VISTA	1747 + 17470	BiRen	NA	0.957	NA	NA	NA	NA
	VISTA	1848 + 18480	FANTOM-ep20	0.946	0.958	0.650	0.975	0.655	0.796

^a^our best pretraining-retraining model by pretraining with large scale FANTOM enhancer data and retraining with 20 epochs; ‘NA’ represents ‘not provided by original publications’.

For comparison with BiRen, we applied our FANTOM-ep20 model on VISTA enhancer data that BiRen used. We visited the updated version of VISTA enhancer browser https://enhancer.lbl.gov/ and downloaded 959 positive human enhancer sequences and 889 negative ones, summing 1,848 human enhancer sequences. To be consistent with BiRen, a non-enhancer dataset containing 10 times the number of random genomic fragments (18,480 non-enhancer sequences) were selected from the whole genome (the GRCh37 reference genome) by excluding exon, intron and known enhancers. As a result, our FANTOM-ep20 model achieved an average AUC value of 0.958, which is slightly larger than 0.957 of BiRen by evaluating *via* a five-fold cross validation test. Moreover, additional evaluation indices including ACC, GM, Sens, and Spec of our FANTOM-ep20 model are also provided in **Table 2**, from which we found that a GM value of 0.796 was achieved, suggesting our FANTOM-ep20 model remains robust prediction performance on VISTA enhancer data.

## Discussion

Enhancers are important CREs and play significant roles in gene transcriptional regulation. Majority of enhancers have strong cell or tissue specificity, which highlights the importance of TSEP. In this paper, we developed a novel training strategy of deep learning named with PRS, which was proven to be a reliable prediction model for TSEP. Finally, we conclude that PRS brings some new contributions or findings into the area of TSEP:

New contribution to training strategy: a specific cell or tissue type has only hundreds or a few thousands of specific enhancer samples, which might make existing deep learning methods to fall into overfitting problem. PRS employs a large scale FANTOM enhancers data to construct a pretraining model with optimal model hyperparameters, and then uses each small sample dataset of tissue-specific enhancers to retrain, based on the trained pretraining model. Testing results on 23 different cell or tissue types demonstrate that PRS is superior to classic training strategy without pretraining, which enable us to conclude that PRS is a reliable method for TSEP.

New findings on optimal retraining epochs: we found that 20 additional epochs are optimal when retraining a new source of tissue-specific enhancer samples based on the trained pretraining model. Either too few or too many additional epochs are not the good choices, because too few epochs like FANTOM-ep10 has not fully learned features of the new source data, whereas too many epochs like FANTOM-ep50 might has a big problem of overfitting.

New contribution to transfer learning: when comparing the best model of PRS named with FANTOM-ep20 with existing tool names with BiRen, we noted an interesting fact: FANTOM-ep20 achieved a greater AUC value with a different enhancer data source of VISTA enhancer data in the retraining stage. VISTA enhancer data was generated with a totally different biological assay and has distinct distribution or source domain with FANTOM enhancer data. Our FANTOM-ep20 model took pretraining with FANTOM enhancer data and then performed retaining with VISTA enhancer data. This shows that our PRS model has good performance of transfer learning, which implies that PRS might provide helpful ideas for transfer learning studies.

Although notable successes were achieved in the current study, some drawbacks or limitations still need further investigations in the future works. For example, this method is not appropriate for enhancers with sequences shorter than 100bp and greater than 1000bp. In addition, there are totally three main sources of enhancer data: FANTOM, Vista, and ENCODE. In the current study, we only trained on FANTOM enhancer data and tested on Vista enhancer data. The comprehensive combinations of training and testing between three sources are the future directions of DEP and TSEP.

## Data Availability Statement

We developed our scripts and pipeline with the “Keras” deep learning framework in Python. We deposited our data, codes, and trained models at the following github website: https://github.com/yangg-kun/enhancer_retraining.

## Author Contributions

XH and XN designed the research. KY, GZ and ZQ performed the research and analyzed the data. XH and XN wrote the manuscript. All authors revised the manuscript.

## Funding

XN was partially supported by the Fundamental Research Funds for the Central Universities HZAU (Grant No. 2662017JC048). XH was partially supported by the National Natural Science Foundation of China (NSFC) (Grant No. 11671003).

## Conflict of Interest

The authors declare that the research was conducted in the absence of any commercial or financial relationships that could be construed as a potential conflict of interest.
