# Supplementary material for: A Pretraining-Retraining Strategy of Deep Learning Improves Cell-Specific Enhancer Predictions
Source: Front Genet. 2020 Jan 8;10:1305. doi: 10.3389/fgene.2019.01305 (PMC6960260; doi:10.3389/fgene.2019.01305)
Supplement: Supplementary file 1 [file DataSheet_1.docx]

Supplementary Materials


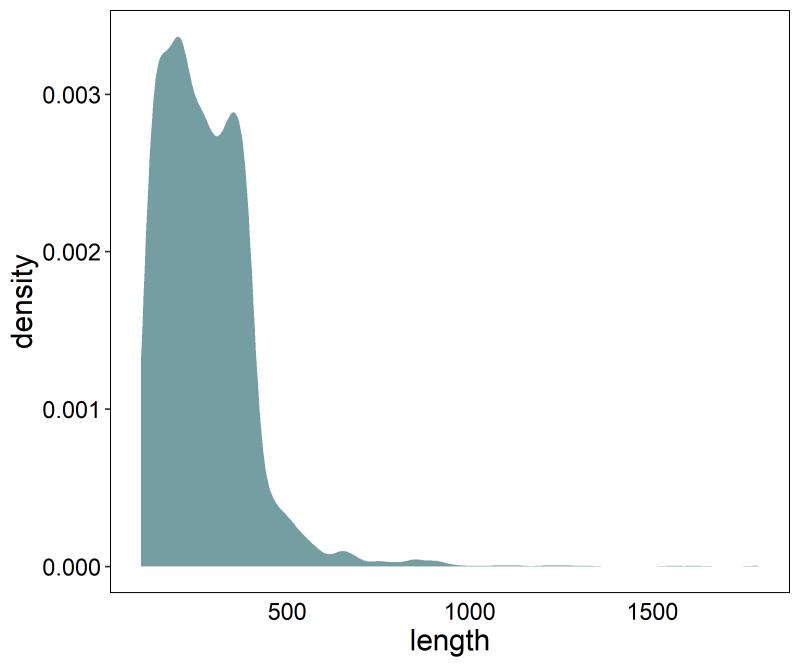


Supplementary Figure 1. The plot of length distribution of 4653 positive enhancer samples.


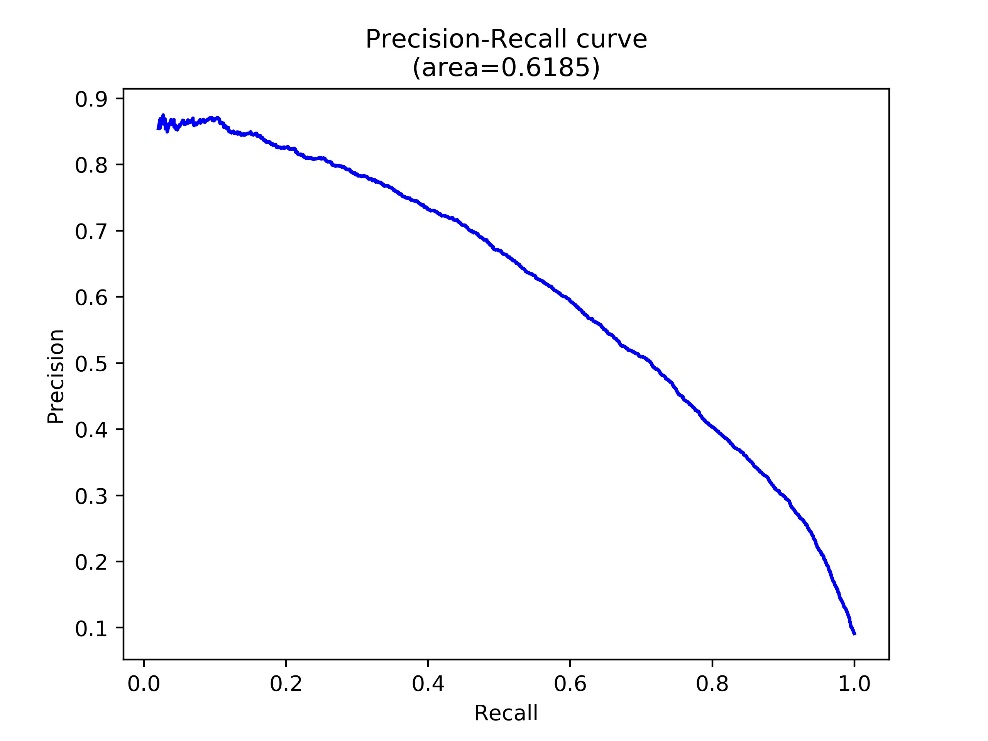


Supplementary Figure 2. The precision-recall curve of the 5-fold-cross-validation test.


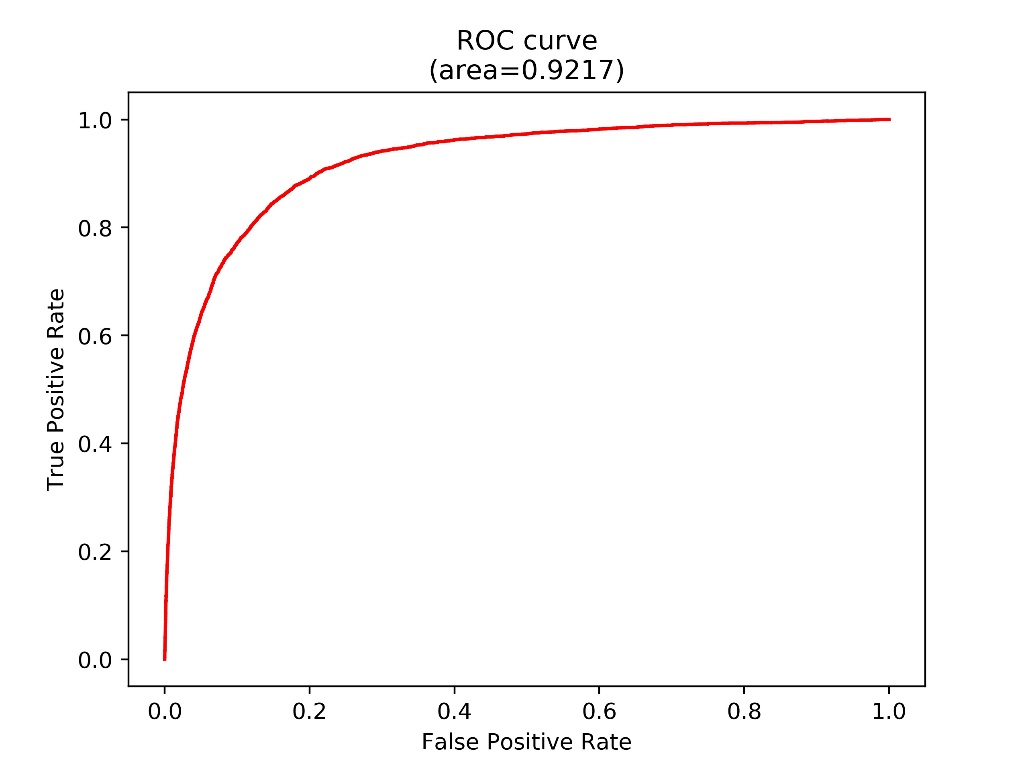


Supplementary Figure 3. The receiver operating characteristic (ROC) curve of the 5-fold-cross-validation test.
